# Supplementary material for: Resolving the full spectrum of human genome variation using Linked-Reads
Source: Genome Res. 2019 Apr;29(4):635–45. doi: 10.1101/gr.234443.118 (PMC6442396; doi:10.1101/gr.234443.118)
Supplement: Supplemental Material [file supp_gr.234443.118_Supplemental_longranger-2.2.zip › longranger-2.2.tar.bz2!/lr2.2-public/lib/stan/cmdstan/stan/doc/api/html/fixed__param__sampler_8hpp_source.html]

Stan: src/stan/mcmc/fixed\_param\_sampler.hpp Source File


|  |  |
| --- | --- |
|  | Stan  2.5.0  probability, sampling & optimization |


- Main Page
- Related Pages
- Namespaces
- Classes
- Files

- File List
- File Members

All Classes Namespaces Files Functions Variables Typedefs Enumerations Enumerator Friends Macros Pages

- src
- stan
- mcmc

fixed\_param\_sampler.hpp

Go to the documentation of this file.

1 #ifndef STAN\_\_MCMC\_\_FIXED\_\_PARAM\_\_SAMPLER\_\_HPP

2 #define STAN\_\_MCMC\_\_FIXED\_\_PARAM\_\_SAMPLER\_\_HPP

3

4 #include <iostream>

5 #include <string>

6

7 #include <stan/mcmc/base\_mcmc.hpp>

8 #include <stan/mcmc/sample.hpp>

9

10 namespace stan {

11

12  namespace mcmc {

13

14  class fixed\_param\_sampler: public base\_mcmc {

15

16  public:

17

18  fixed\_param\_sampler(std::ostream\* o = &std::cout, std::ostream\* e = 0):

19  base\_mcmc(o, e) { this->name\_ = "Fixed Parameter Sampler"; }

20

21  sample transition(sample& init\_sample) { return init\_sample; }

22

23

24  };

25

26  } // mcmc

27

28 } // stan

29

30 #endif

31

stan::mcmc::base\_mcmc::name\_

std::string name\_

**Definition:** base\_mcmc.hpp:38

stan::mcmc::fixed\_param\_sampler::transition

sample transition(sample &init\_sample)

**Definition:** fixed\_param\_sampler.hpp:21

base\_mcmc.hpp

stan::mcmc::fixed\_param\_sampler::fixed\_param\_sampler

fixed\_param\_sampler(std::ostream \*o=&std::cout, std::ostream \*e=0)

**Definition:** fixed\_param\_sampler.hpp:18

stan::mcmc::fixed\_param\_sampler

**Definition:** fixed\_param\_sampler.hpp:14

sample.hpp

stan::math::e

double e()

Return the base of the natural logarithm.

**Definition:** constants.hpp:86

stan::mcmc::sample

**Definition:** sample.hpp:13

stan::mcmc::base\_mcmc

**Definition:** base\_mcmc.hpp:13


---

[ Stan Home Page ]

*© 2011–2014,
Stan Development Team.*
